# Neuro-immune-vascular-stem cell crosstalk in bone/cartilage regeneration: mechanisms, technological advances, and clinical perspectives

**DOI:** 10.3389/fbioe.2026.1778578

**Published:** 2026-04-01

**Authors:** Zhichao Liu, Haiyan Fan, Yun Yang

**Affiliations:** 1 Graduate School, Inner Mongolia Medical University, Hohhot, Inner Mongolia, China; 2 Imaging Center, Affiliated Hospital of Inner Mongolia Medical University, Hohhot, Inner Mongolia, China; 3 Center for Joint Surgery, The Second Affiliated Hospital of Inner Mongolia Medical University, Hohhot, Inner Mongolia, China

**Keywords:** angiogenesis, bone marrow mesenchymal stem cells (BMSCs), bone regeneration, cartilage regeneration, clinical translation, endothelial cells, mesenchymal stem cells (MSCs), microphysiological systems (MPSs)

## Abstract

Functional regeneration of bone and cartilage remains an urgent clinical challenge in orthopedics, as its repair process involves the synergistic participation of multiple systems and cell types. Traditional studies have mostly focused on the regulatory roles of individual cells or signaling pathways, while recent research has confirmed that bone/cartilage regeneration is governed by a regulatory mechanism centered on the neuro-immune-vascular axis. In this mechanism, mesenchymal stem cells (MSCs), bone marrow mesenchymal stem cells (BMSCs), adipose-derived mesenchymal stem cells (ADSCs), and cartilage progenitor cells (CPCs) serve as key functional cells, interacting sequentially and transcellularly with immune cells and endothelial cells through multiple core signaling pathways. This review systematically summarizes these core signaling pathways, including neurosignal-mediated pathways (CGRP/*CRLR*, *NGF*/*TrkA*, SP/*NK1R*), immune signal-mediated pathways (*IL-4*/*IL-4R*, *TGF-β*/Smad, *TNF-α*/*NF-κB*), endothelial cell-mediated pathways (*VEGF*/*VEGF*R, Notch, *PDGF*/*PDGF*R), and cross-regulatory pathways (*PI3K*/*Akt*, *MAPK*). These pathways collectively mediate the sequential crosstalk and functional coordination among the four cellular components. Additionally, the review highlights the application achievements of cutting-edge technologies in this field, such as single-cell omics, organoid models, *in vivo* imaging, new approach methodologies (NAM), microphysiological systems (MPSs), and biosensor-integrated platforms. It thoroughly analyzes the current bottlenecks in network mechanism research and clinical translation, including the spatiotemporal specificity of regulatory targets and the difficulty in simulating complex microenvironments, while proposing bre*Akt*hrough directions such as optimizing targeted regulatory strategies, developing intelligent biomaterials, and integrating multi-disciplinary technologies. Notably, the traditional M1/M2 macrophage dichotomy can no longer capture the high heterogeneity of immune cells. Recent single-cell omics studies have identified multiple functionally distinct macrophage subsets in the bone/cartilage regeneration microenvironment. This discovery provides a new perspective for precise immune regulation strategies and also underscores the limitations of the traditional classification framework. Overall, this review aims to establish a systematic framework for understanding the complex regulatory mechanisms of bone/cartilage regeneration and offer theoretical support and research insights for the development of efficient repair strategies.

## 1 Introduction

As core components of the musculoskeletal system, bone and cartilage bear weight, transmit mechanical forces, and protect internal organs. Bone possesses limited self-healing capacity, but repair of large bone defects (e.g., post-traumatic or post-tumor resection) remains trapped in a vicious cycle of “delayed vascularization and insufficient osteogenesis” (Fang et al., 2024). In contrast, cartilage lacks blood vessels, nerves, and lymphatic tissues, resulting in nearly no spontaneous repair after injury and a high risk of progressing to osteoarthritis (Fi et al., 2024). Statistics indicate that tens of millions of patients worldwide require clinical intervention for bone/cartilage injuries annually, imposing a heavy burden on healthcare systems (Fang et al., 2022).

Over the past decades, research on bone/cartilage regeneration has evolved from “single-cell transplantation” to “material-cell-cytokine synergy,” yet clinical translation outcomes remain unsatisfactory. For instance, transplantation of MSCs—particularly bone marrow mesenchymal stem cells (BMSCs)—exhibits promising osteogenic/chondrogenic potential in animal studies, but their survival and differentiation efficiency decline significantly in clinical settings due to inadequate microenvironmental support (Han et al., 2025). The aforementioned microenvironmental deficiencies involve multiple critical factors: mismatched biocompatibility and degradation kinetics of scaffold design, inadequate targeted delivery and spatiotemporal concentration regulation of biochemical signaling molecules, insufficient nutritional supply stemming from delayed angiogenesis, inflammatory interference induced by an imbalanced immune microenvironment, as well as remarkable discrepancies between *in vitro* culture conditions and the intricate *in vivo* physiological microenvironment (e.g., mechanical stimulation, the integrity of intercellular crosstalk networks, etc.).

In recent years, with the integration of neurobiology, immunology, and tissue engineering, researchers have recognized that bone and cartilage are not “isolated” organs. Their regeneration is precisely regulated by the nervous and immune systems: the nervous system not only directly modulates stem cell proliferation and differentiation but also shapes a regeneration-permissive immune microenvironment by regulating the activation state, polarization direction, and cytokine secretion profile of immune cells. Immune cells, acting as “responders” to neural signals and “executors” of stem cell regulation, form the core regulatory axis of the neuro-immune-stem cell network (Dalle Carbonare et al., 2025). The proposal of this cross-system regulatory theory provides a new perspective for overcoming bottlenecks in bone/cartilage regeneration. Notably, the synergy between bone regeneration and angiogenesis is a prerequisite for successful repair, with endothelial cells acting as “bidirectional regulators”: On one hand, endothelial cells directly modulate the osteogenic potential of MSCs and BMSCs and induce macrophage polarization toward “factor-secreting” subsets via secreting cytokines such as *VEGF*, *PDGF*, and fibroblast growth factor (FGF), enhancing the immunomodulatory repair microenvironment. On the other hand, they form functional vascular networks to deliver oxygen, nutrients, and remove metabolic waste, addressing the critical issue of “central necrosis” in large bone defect repair. Additionally, endothelial cells mediate adhesion and recruitment of immune cells and stem cells via expressing intercellular adhesion molecule-1 (*ICAM-1*) and vascular cell adhesion molecule-1 (*VCAM-1*), promoting direct intercellular communication. Thus, the neuro-immune-stem cell network does not act independently but forms a multi-dimensional regulatory network with the vascular system—the “neuro-immune-vascular-stem cell network”—where the four components interact via complex signal crosstalk and temporal synergy to jointly regulate bone/cartilage regeneration.

This review will systematically elaborate on the research progress of this regulatory network from three core aspects: first, dissecting the core interaction mechanisms and key signaling pathways among nerves, immune cells, stem cells, and endothelial cells, with an emphasis on the central role and molecular mechanisms of the neuro-immune-vascular regulatory axis; second, summarizing the application value of cutting-edge technologies such as single-cell omics, organoids, NAM, and MPSs in revealing network regulatory rules, focusing on the resolution of immune cell heterogeneity and challenges to the traditional M1/M2 classification framework by single-cell omics; finally, analyzing current challenges in clinical translation and proposing future development directions, aiming to provide comprehensive references for research and application in this field.

## 2 Core components and interaction basis of the “neuro-immune-stem cell” network (including endothelial cell regulation)

### 2.1 Biological characteristics of core network cells

The core components of the “neuro-immune-stem cell” network include three types of functional cells: nerve cells (and terminals), immune cells (centered on macrophages), and stem cells (represented by MSCs, BMSCs, adipose-derived mesenchymal stem cells (ADSCs), and cartilage progenitor cells (CPCs)). Additionally, endothelial cells, as key nodes in angiogenesis and bone-immune regulation, have become indispensable core components of the network. These four cell types exhibit distinct spatial distribution and functional division in the bone/cartilage regenerative microenvironment, collectively maintaining the orderly progression of regeneration.

#### 2.1.1 Nerve cells and terminals: “dual initiators” of immune regulation and regenerative signals

Bone is a highly innervated organ, with nerve terminals primarily derived from sympathetic, sensory, and peptidergic nerves, widely distributed in the periosteum, Haversian canals, and bone marrow cavity. Although cartilage itself lacks innervation, nerve terminals in the subchondral bone and surrounding synovial tissue can act on cartilage injury sites via secreting neurotransmitters (Grässel and Muschter, 2017). The signaling molecules secreted by these nerve terminals mainly fall into two categories: classical neurotransmitters (e.g., norepinephrine, acetylcholine) and neurotrophic factors [e.g., nerve growth factor (*NGF*), brain-derived neurotrophic factor (BDNF)] and neuropeptides [e.g., calcitonin gene-related peptide (CGRP), substance P (SP)] (Cai and Khoutorsky, 2025).

The core function of nerve cells and terminals is not only to initiate regenerative signals but also to precisely regulate the recruitment, polarization, and functional state of immune cells, constructing a regeneration-permissive immune microenvironment: In the early stage of bone defect, CGRP released by sensory nerve terminals rapidly recruits immune cells to the injury site while inhibiting excessive activation of pro-inflammatory immune cells. Norepinephrine released by sympathetic nerves regulates the polarization direction of immune cells, promoting the phenotypic switch of repair-related immune cells, collectively initiating the construction of the regenerative microenvironment (Park et al., 2024). Furthermore, nerve terminals modulate the cytokine secretion profile of immune cells via secreting factors such as BDNF and *NGF*, further strengthening the neuro-immune regulatory axis.

Notably, direct signal crosstalk exists between nerve terminals and endothelial cells: CGRP released by sensory nerves activates the *CRLR* receptor on endothelial cells, promoting *VEGF* secretion and endothelial cell proliferation/migration, accelerating vascular network formation. Norepinephrine released by sympathetic nerves inhibits excessive endothelial cell activation via α1 receptors, avoiding abnormal vascular proliferation, forming an initial loop of “neuro-endothelial” synergistic regulation of angiogenesis.

#### 2.1.2 Immune cells: “Responders” to neural signals and “hubs” of stem cell regulation

Immune cells are core regulatory cells in the bone/cartilage regenerative microenvironment, with macrophages serving as “key nodes” of network regulation due to their high plasticity. Traditional studies classify macrophages into M1 (pro-inflammatory) and M2 (anti-inflammatory/repair) phenotypes, but this dichotomy has been significantly challenged. Recent single-cell omics studies confirm that macrophages in the bone/cartilage regenerative microenvironment do not undergo simple bipolar differentiation but exist as multiple functionally distinct subsets (e.g., “inflammation-regulating,” “factor-secreting,” “phagocytic,” “vascular-associated”) (Li J. et al., 2025; Wang et al., 2024). These subsets exhibit significant differences in surface markers, cytokine secretion profiles, and functional division: For example, “inflammation-regulating” macrophages highly express CGRP receptor (*CRLR*) and adrenergic receptors, directly responding to neural signal regulation and maintaining the dynamic balance of the inflammatory microenvironment via rapid functional switching. “Factor-secreting” macrophages highly express repair factors such as *IL-4*, *TGF-β*, and *PDGF*, with their infiltration peak highly synchronized with stem cell differentiation, serving as the core link connecting neural signals and stem cell functions (Wang et al., 2024).

Among these, the interaction between “vascular-associated” macrophages and endothelial cells is particularly critical: This subset highly expresses *VEGF* receptor (*VEGFR1*) and *PDGF*-B, promoting endothelial cell proliferation via paracrine *PDGF*-B and mediating endothelial cell tube formation via direct contact. In turn, IL-6 secreted by endothelial cells maintains the phenotypic stability of “vascular-associated” macrophages.

Despite the widespread use of the traditional M1/M2 classification framework in biomaterials research, its limitations have become increasingly prominent: It fails to cover the heterogeneous characteristics of macrophages, ignoring intermediate phenotypes and functional subsets. Moreover, macrophage phenotypic switching *in vivo* is regulated by multiple factors such as neural signals, cytokines, and mechanical stimulation, rather than a simple “either/or” model (Oliver et al., 2024). Beyond macrophages, neutrophils and T cell subsets (e.g., Th17 cells, Treg cells) also participate in constructing the neuro-immune regulatory axis: Neutrophils recruit nerve terminals and macrophages via releasing chemokines in the early stage of injury, forming an early regulatory loop of “neuro-neutrophil-macrophage.” IL-10 secreted by Treg cells enhances the stability of repair-related macrophages and responds to neuropeptide CGRP regulation, further strengthening the synergy of the neuro-immune-stem cell network (Chen et al., 2022). Additionally, neutrophils degrade the basement membrane via releasing matrix metalloproteinase (*MMP-9*), creating space for endothelial cell migration and indirectly promoting angiogenesis. IL-10 secreted by Treg cells inhibits endothelial cell inflammatory responses, reducing the expression of adhesion molecules and avoiding excessive immune cell infiltration-induced damage to the vascular network.

#### 2.1.3 Stem cells: “executors” of tissue regeneration and “feedback regulators” of the network

Stem cells are core functional cells for bone/cartilage regeneration, with BMSCs, ADSCs, and CPCs widely used due to their easy accessibility and stable differentiation potential. The osteogenic/chondrogenic differentiation of stem cells is precisely regulated by neuro-immune signals: They express various neurotransmitter receptors (e.g., CGRP receptor, adrenergic receptor) and immune factor receptors (e.g., *IL-4* receptor, *TGF-β* receptor) on their surface, enabling them to directly “sense” changes in neuro-immune signals and adjust their differentiation direction (Li et al., 2010). For example, activation of the *NGF* receptor *TrkA* on BMSCs enhances their osteogenic differentiation potential via the *PI3K*/*Akt* pathway. Binding of *IL-4* to *IL-4R* on BMSCs significantly upregulates the expression of the cartilage-specific gene *COL2A1* (Hao et al., 2024). Furthermore, the *NGF-TrkA* signal activates the *MAPK*/ERK pathway, which has been confirmed to promote BMSCs proliferation and neuron-like differentiation.

Notably, *NGF* exhibits dual functions: On one hand, it acts as a cartilage protector and osteogenesis promoter, participating in regeneration via regulating stem cell differentiation and cell balance. On the other hand, *NGF* is closely associated with pain sensitivity, and its overexpression may exacerbate pain at the injury site. For instance, in osteoarthritis models, abnormal elevation of *NGF* may delay degeneration via promoting CPC differentiation or trigger chronic pain via activating sensory nerves. This contradictory effect requires further regulation based on intervention timing and concentration.

A “bidirectional empowerment” regulatory relationship exists between stem cells and endothelial cells: On one hand, BMSCs promote endothelial cell proliferation, migration, and tube formation via secreting factors such as *VEGF* and *FGF-2*. They also activate the Notch signaling pathway via expressing *Jagged1* (binding to *Notch1* on endothelial cells), maintaining the stability of the vascular network. On the other hand, endothelial cells directly regulate BMSCs differentiation into osteoblasts via secreting factors such as bone morphogenetic protein (*BMP-2*) and *TGF-β*. The vascular microenvironment constructed by endothelial cells significantly improves the survival efficiency of BMSCs at the injury site. Additionally, ADSCs promote the expression of *VEGFR2* in endothelial cells via paracrine exosomes carrying *miR-126*, enhancing angiogenesis and indirectly improving osteogenic outcomes.

#### 2.1.4 Endothelial cells: “central hubs” of angiogenesis and bone-immune regulation

As key regulatory cells in the bone/cartilage regenerative microenvironment, endothelial cells are widely distributed in periosteal blood vessels, Haversian canals, and bone marrow microvessels. Their core functions are reflected in three dimensions: angiogenesis, immune regulation, and osteogenic synergy.

1. Angiogenic function: Endothelial cells form functional vascular networks via proliferation, migration, and tube formation, delivering oxygen, nutrients (e.g., amino acids, glucose), and signaling molecules to regenerating tissues while removing metabolic waste. This is critical for addressing “central necrosis” in large bone defect repair. In the early stage of bone regeneration, endothelial cells rapidly respond to injury signals, initiating angiogenesis via the *VEGF*-*VEGFR2* pathway to form initial vascular plexuses. In the remodeling stage, they interact with pericytes and “vascular-associated” macrophages to promote vascular maturation and stability.
2. Immunomodulatory function: Endothelial cells regulate the recruitment and polarization of immune cells via expressing adhesion molecules (*ICAM-1*, *VCAM-1*) and secreting cytokines (IL-6, IL-8, *TGF-β*): In the early stage of injury, endothelial cells highly express *ICAM-1*, mediating the adhesion and infiltration of neutrophils and monocytes. In the later stage, they secrete *TGF-β* to inhibit the release of pro-inflammatory factors, promoting macrophage polarization toward “factor-secreting” subsets. Additionally, endothelial cells inhibit excessive immune responses and maintain the homeostasis of the regenerative microenvironment via expressing programmed death ligand 1 (*PD-L1*), which binds to *PD-1* on T cells.
3. Osteogenic synergy function: Factors such as *BMP-2*, *VEGF*, and *PDGF* secreted by endothelial cells directly regulate stem cell osteogenic differentiation. Direct cell-cell contact between endothelial cells and osteoblasts also plays a regulatory role: *E-cadherin* on endothelial cells binds to *N-cadherin* on osteoblasts, promoting osteoblast proliferation and mineralization. Studies confirm that endothelial cell-specific knockout of the *VEGF* gene leads to insufficient vascularization and osteogenesis in bone defect repair, while supplementation with exogenous *VEGF* partially restores repair outcomes, directly verifying the central role of endothelial cells in “vascularization-osteogenesis” synergy.

### 2.2 Temporal patterns of network interactions: three-stage progression of neuro-immune-vascular-stem cell synergy

**FIGURE 1:**
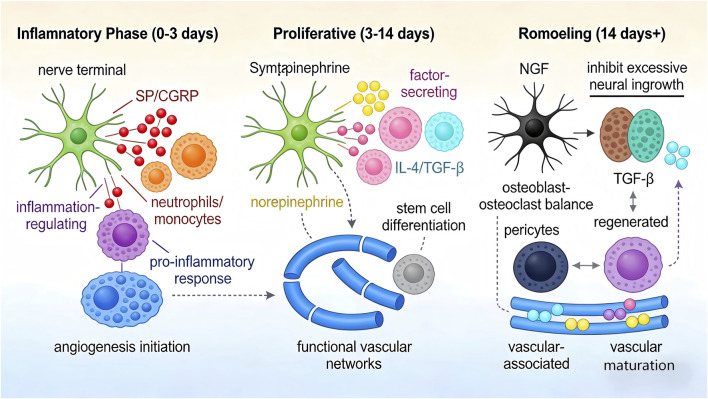
Schematic diagram of the temporal interaction patterns of the “neuro-immune-vascular-stem cell” network. The figure clearly shows the core functions, key signaling molecules, and interaction relationships of nerve cells, immune cells, endothelial cells, and stem cells in the three regeneration stages. Inflammatory phase (0–3 days): Nerve terminals release SP/CGRP, recruiting neutrophils/monocytes; “inflammation-regulating” macrophages dominate pro-inflammatory responses; endothelial cells initiate angiogenesis. Proliferative phase (3–14 days): Sympathetic nerves release norepinephrine; macrophages polarize toward “factor-secreting” subsets, releasing *IL-4*/*TGF-β*; endothelial cells form functional vascular networks, synergistically inducing stem cell differentiation. Remodeling phase (14 days+): *NGF* regulates osteoblast-osteoclast balance; *TGF-β* inhibits excessive neural ingrowth; endothelial cells cooperate with pericytes and “vascular-associated” macrophages to maintain vascular maturation, ensuring the stability of regenerated tissues. Arrows in the figure indicate signal transmission directions, and different colors distinguish cell types and signaling molecules.

The interactions within the “neuro-immune-stem cell” network exhibit strict temporal specificity, highly synchronized with the three stages of bone/cartilage regeneration—“inflammatory phase, proliferative phase, remodeling phase.” The neuro-immune-vascular regulatory axis plays a dominant role in each stage, and its dysfunction is the core cause of regeneration failure (Figure 1).

#### 2.2.1 Inflammatory phase (0–3 days post-injury)

Injury signals first activate local nerve terminals, with sensory nerves releasing SP and CGRP: SP recruits neutrophils and monocytes to the injury site via activating the *NK1R* pathway, forming early inflammatory infiltration. CGRP regulates the differentiation direction of monocytes via the *CRLR* pathway, inhibiting their excessive switch to pro-inflammatory phenotypes (Chen et al., 2024). At this stage, “inflammation-regulating” macrophages dominate, clearing necrotic tissue via releasing pro-inflammatory factors such as *TNF-α* and IL-6, while expressing neural signal receptors to form a bidirectional regulatory loop of “neuro-macrophage” (Wang et al., 2024).

Simultaneously, injury signals activate endothelial cells, which rapidly secrete *VEGF* and *MMP-9*: *VEGF* promotes endothelial cell proliferation and migration, while *MMP-9* degrades the extracellular matrix to create space for angiogenesis. Endothelial cells upregulate the expression of *ICAM-1* and *VCAM-1*, mediating the adhesion and infiltration of immune cells, forming an early regulatory network of “neuro-immune-endothelial.” The core feature of this stage is “neural initiation of immunity and angiogenesis, immune shaping of the inflammatory microenvironment, and endothelial construction of initial nutrient pathways,” with the three components synergistically laying the foundation for subsequent regeneration.

#### 2.2.2 Proliferative phase (3–14 days post-injury)

Norepinephrine released by sympathetic nerves and IL-1β secreted by M1 macrophages collectively regulate macrophage polarization toward “factor-secreting” subsets. These subsets release *IL-4*, IL-10, and *TGF-β*, which not only inhibit excessive inflammatory responses but also induce stem cell differentiation into osteoblasts/chondrocytes via activating signaling pathways on the stem cell surface (Wang et al., 2024). Meanwhile, BDNF secreted by nerve terminals promotes the survival and functional stability of macrophages, reducing the apoptosis of repair-related macrophages, forming a regulatory chain of “neuro-macrophage-stem cell” (Wang et al., 2024).

The core role of endothelial cells at this stage is to construct functional vascular networks: Endothelial cells form tube structures under the action of factors such as *VEGF* and *PDGF*, interacting with “vascular-associated” macrophages and pericytes to promote vascular maturation. The mature vascular network provides sufficient nutrients and oxygen to stem cells, significantly improving their differentiation efficiency. Additionally, endothelial cells secrete *BMP-2* and *TGF-β*, which synergize with *IL-4* secreted by macrophages to enhance stem cell osteogenic/chondrogenic differentiation, forming a multi-cell regulatory loop of “neuro-immune-endothelial-stem cell.” The core feature of this stage is “neural regulation of immune phenotypic switching, endothelial construction of nutrient pathways, and immune-endothelial synergistic guidance of stem cell differentiation,” with the four components jointly driving the regeneration process.

#### 2.2.3 Remodeling phase (14 days post-injury to months)

Neuro-immune signals gradually weaken but remain critically regulatory: *NGF* secreted by nerve terminals promotes the mineralization and remodeling of bone matrix via regulating the balance between osteoblasts and osteoclasts. “Vascular-associated” macrophages cooperate with endothelial cells and nerve terminals to maintain the balance between vascularization and innervation of regenerated tissues (Li et al., 2025a). *TGF-β* autocrine by chondrocytes inhibits excessive neural ingrowth, preventing neural fibers from invading cartilage tissue and affecting its function (Wang T. et al., 2025).

Specifically, the regulatory mechanism of *NGF* on osteoblast-osteoclast balance involves two pathways: First, *NGF* binds to the *TrkA* receptor on osteoblasts, promoting osteoblast proliferation, mineralization, and the expression of osteogenic-specific genes (*Runx2*, *OCN*) via activating the *PI3K*/*Akt* and *MAPK* pathways. Second, *NGF* binds to the *p75NTR* receptor on osteoclasts, inhibiting the differentiation of osteoclast precursors into mature osteoclasts and promoting the apoptosis of mature osteoclasts, thereby reducing bone resorption (Kadota-Watan et al., 2025). In cartilage remodeling, the *NGF*/*TrkA* pathway inhibits chondrocyte dedifferentiation, maintaining their ability to secrete type II collagen. *TGF-β* downregulates the expression of neural growth-related genes (e.g., *GAP-43*) via the Smad pathway, preventing abnormal invasion of neural fibers into cartilage tissue (Wang T. et al., 2025).

The core function of endothelial cells at this stage is to maintain vascular network stability and osteogenesis-vascular synergy: Endothelial cells closely bind to pericytes, inhibiting excessive endothelial cell proliferation via the Notch signaling pathway to avoid abnormal vascular proliferation. *PDGF*-BB secreted by endothelial cells promotes pericyte recruitment and vascular wall maturation, ensuring the structural stability of the vascular network. Additionally, endothelial cells interact with osteoblasts via direct contact and paracrine signals, promoting bone matrix mineralization and ensuring the matching between the mechanical properties of regenerated bone and the degree of vascularization. The core feature of this stage is “neuro-immune-vascular-stem cell synergy maintaining the structural and functional maturation of regenerated tissues,” with vascular network stability being critical for the long-term survival of regenerated tissues.

This strict temporal regulation ensures the orderly progression of bone/cartilage regeneration. Dysfunction of the neuro-immune-vascular regulatory axis is the key to regeneration failure: For example, insufficient endothelial cell activation during the inflammatory phase leads to delayed angiogenesis, nutrient deprivation, and inhibited stem cell survival. Insufficient polarization of “vascular-associated” macrophages during the proliferative phase results in impaired vascular maturation and reduced stem cell osteogenic differentiation efficiency. Unstable vascular networks during the remodeling phase lead to degenerative changes in regenerated tissues, ultimately forming fibrous repair tissue (Liu H. et al., 2025).

## 3 Analysis of core regulatory pathways in the “neuro-immune-stem cell” network (including endothelial cell-related pathways)

Interactions among nerves, immune cells, and stem cells are mediated by a series of core signaling pathways, which are both independent and interconnected, forming a complex regulatory network. Additionally, endothelial cells participate in network regulation via multiple specific pathways, forming cross-links with neural, immune, and stem cell signals to constitute the “neuro-immune-vascular-stem cell” pathway network. Among these, pathways related to the neuro-immune-vascular regulatory axis are the core hubs of the network: Neural signals regulate the functions of immune cells and endothelial cells, indirectly affecting stem cell differentiation, forming a three-level regulatory chain of “neural signal → immune cell/endothelial cell → stem cell.” Based on the source of signals, these pathways can be classified into four categories: “neural signal-mediated immune and endothelial regulatory pathways,” “immune signal-mediated stem cell and endothelial regulatory pathways,” “endothelial signal-mediated osteogenic and immune regulatory pathways,” and “shared cross-regulatory pathways among the four components.”

### 3.1 Neural signal-mediated core regulatory pathways: focus on immune cell and endothelial cell function regulation

Neurotransmitters, neuropeptides, and neurotrophic factors secreted by nerve terminals activate signaling pathways in immune cells, endothelial cells, and stem cells via specific receptors. Among these, the synergistic regulation of immune cells and endothelial cells is the core pathway through which neural signals participate in regeneration, with the CGRP/*CRLR*, *NGF*/*TrkA*, and SP/*NK1R* pathways being the most critical.

#### 3.1.1 CGRP/*CRLR* pathway: “Core regulator” of immune polarization, “initiator” of endothelial activation, and “promoter” of stem cell proliferation

CGRP is a core neuropeptide secreted by sensory nerves, with its receptor *CRLR* widely expressed on macrophages, neutrophils, T cells, MSCs, and endothelial cells. This pathway plays a key role in constructing the neuro-immune-vascular regulatory axis:

- In immune cells: Binding of CGRP to *CRLR* inhibits the nuclear translocation of *NF-κB* via activating the cAMP/PKA pathway, thereby suppressing the activation of pro-inflammatory macrophages (traditional M1 type) and the release of pro-inflammatory factors such as *TNF-α* and IL-6, while promoting macrophage polarization toward “factor-secreting” subsets (traditional M2 type) (Duan et al., 2017). Studies confirm that CGRP gene knockout in bone defect models leads to a significant increase in the proportion of pro-inflammatory macrophages at the injury site, prolonged inflammatory response, insufficient infiltration of “factor-secreting” macrophages, and ultimately reduced stem cell osteogenic differentiation efficiency (Chen et al., 2024). Additionally, CGRP regulates the proliferation and function of Treg cells, further strengthening the construction of an immunosuppressive microenvironment via enhancing their ability to secrete IL-10.
- In endothelial cells: Binding of CGRP to *CRLR* promotes endothelial cell proliferation and migration via activating the cAMP/PKA pathway, while upregulating the expression of *VEGF* and *eNOS. eNOS* catalyzes the production of NO, which dilates blood vessels and inhibits platelet aggregation, creating a favorable environment for angiogenesis. Studies have found that local application of CGRP significantly increases the vascular density in bone defect areas, promotes vascular network maturation, and enhances osteogenic outcomes, while *CRLR* inhibitors reverse this effect.
- In stem cells: The CGRP/*CRLR* pathway promotes MSCs proliferation via activating ERK1/2 signals and upregulating the expression of Cyclin D1. Simultaneously, this pathway enhances MSCs osteogenic differentiation potential via regulating the activity of *Runx2* (Jiang et al., 2022). Additionally, CGRP indirectly promotes the vascularization of regenerated areas via regulating the proliferation and migration of vascular endothelial cells, forming synergistic regulation with the neuro-immune-stem cell network. Relevant basic studies have also revealed that the regulatory role of the CGRP/*CRLR* pathway in bone metabolism is evolutionarily conserved, existing in similar mechanisms from rodents to primates, providing cross-species theoretical support for clinical translation.

#### 3.1.2 *NGF*/*TrkA* pathway: “stabilizer” of repair-related immune cells, “modulator” of endothelial function, and “regulator” of osteogenic/chondrogenic differentiation

*NGF* is mainly secreted by sympathetic nerve terminals, fibroblasts at the injury site, and “factor-secreting” macrophages. Its high-affinity receptor *TrkA* is expressed on macrophages, BMSCs, CPCs, osteoblasts, and endothelial cells. The regulation of the neuro-immune-vascular regulatory axis by *NGF* is reflected in three aspects:

- In immune cells: Binding of *NGF* to *TrkA* on macrophages inhibits macrophage apoptosis via activating the *PI3K*/*Akt* pathway, while promoting their secretion of repair factors such as *IL-4* and *TGF-β*, enhancing the stability of “factor-secreting” macrophages (Kadota-Watan et al., 2025). Additionally, *NGF* inhibits the excessive activation of neutrophils, reducing the release of myeloperoxidase (MPO) and avoiding inflammatory damage to regenerated tissues.
- In endothelial cells: Binding of *NGF* to *TrkA* promotes endothelial cell proliferation, migration, and tube formation via activating the *PI3K*/*Akt*/*eNOS* pathway, while upregulating the expression of *VEGFR2*, enhancing the sensitivity of endothelial cells to *VEGF*. However, high concentrations of *NGF* may induce endothelial cell apoptosis via activating the *p75NTR* receptor, leading to vascular network destruction, thus its role is concentration-dependent.
- In stem cells: Binding of *NGF* to *TrkA* inhibits stem cell apoptosis via the *PI3K*/*Akt* pathway, while activating the downstream *MAPK* pathway, upregulating the expression of osteogenic-specific genes (*Runx2*, *OCN*) and cartilage-specific genes (*COL2A1*, *Aggrecan*) (Liu J. et al., 2025). During bone remodeling, *NGF* also regulates the balance between osteoblasts and osteoclasts: On one hand, it promotes osteoblast proliferation and mineralization via the *TrkA* pathway. On the other hand, it binds to the *p75NTR* receptor on osteoclasts, inhibiting osteoclast activation (Kadota-Watan et al., 2025). In cartilage regeneration, the *NGF*/*TrkA* pathway significantly inhibits chondrocyte dedifferentiation, maintaining their ability to secrete type II collagen and avoiding the formation of fibrocartilage (Wang M. et al., 2025).

#### 3.1.3 SP/*NK1R* pathway: “initiator” of early inflammation, “inducer” of endothelial recruitment, and “guide” of immune-stem cell recruitment

SP is a key neuropeptide released by sensory nerves in the early stage of injury, with its receptor *NK1R* mainly expressed on neutrophils, monocytes, macrophages, MSCs, and endothelial cells. The SP/*NK1R* pathway is the core pathway through which nerves initiate early immune responses and angiogenesis:

- In immune cells: Binding of SP to *NK1R* promotes immune cells (mainly monocytes and neutrophils) to release chemokines (e.g., monocyte chemoattractant protein-1 (*MCP-1*), interleukin-8 (*CXCL8*)) via activating the PLC-γ/IP3 pathway, enhancing the chemotactic ability of neutrophils and monocytes, and accelerating inflammatory infiltration at the injury site [Spitsin et al., 2017]. In the early stage of bone defect, SP release rapidly recruits monocytes to the injury site, promoting their differentiation into “inflammation-regulating” macrophages to clear necrotic tissue (Jiang et al., 2012).
- In endothelial cells: Binding of SP to *NK1R* promotes endothelial cells to secrete *MCP-1* and *CXCL8* via activating the PLC-γ/IP3/Ca^2+^ pathway, further recruiting neutrophils and monocytes, while promoting endothelial cell proliferation and migration, accelerating angiogenesis. Studies confirm that SP-pretreated endothelial cells form more mature tube structures *in vitro*, while the vascular density in bone defect areas of *NK1R* knockout mice is significantly reduced, with impaired repair outcomes.
- In stem cells: SP promotes MSCs migration to the injury site via activating *NK1R* on MSCs and upregulating the expression of migration-related genes (e.g., *CXCR4*) (Tao et al., 2022).

Notably, the role of SP is concentration-dependent: Low concentrations of SP initiate moderate inflammatory responses and angiogenesis, promoting the activation of the neuro-immune-vascular-stem cell network. High concentrations of SP excessively activate inflammatory responses, leading to the massive release of pro-inflammatory factors and aggravating tissue damage (Li et al., 2022).

### 3.2 Immune signal-mediated core regulatory pathways: “dual regulators” of stem cell differentiation and endothelial function

Cytokines secreted by immune cells are the core signal carriers of the “neuro-immune-stem cell” network, whose functions depend on the pre-regulation of immune cell phenotypes by neural signals—i.e., neural signals determine the cytokine secretion profile of immune cells, which then regulate stem cell differentiation and endothelial cell function via immune factors. Among these, signaling pathways mediated by *IL-4*, IL-10, and *TGF-β* secreted by M2 macrophages (“factor-secreting” subsets) play a dominant role in regulating stem cell differentiation and endothelial function. *TNF-α* and IL-6 secreted by M1 macrophages (“inflammation-regulating” subsets) are mainly involved in early inflammatory initiation and endothelial cell activation.

#### 3.2.1 *IL-4*/*IL-4R* pathway: “inducer” of chondrogenic differentiation, “enhancer” of immunosuppression, and “modulator” of endothelial stability

*IL-4* is a core anti-inflammatory factor secreted by “factor-secreting” macrophages, with its receptor *IL-4R* composed of α and γ chains, widely expressed on MSCs, chondrocytes, and endothelial cells. The secretion of *IL-4* is strictly regulated by neural signals: Neuropeptides such as CGRP and *NGF* significantly upregulate *IL-4* expression via activating receptors on macrophages, forming a regulatory chain of “neural signal → macrophage → *IL-4* → stem cell/endothelial cell” (Callejas et al., 2025).

- In stem cells: Binding of *IL-4* to *IL-4R* upregulates the expression of the cartilage-specific transcription factor *Sox9* via activating the JAK1/STAT6 pathway. *Sox9* directly binds to the promoter regions of cartilage-specific genes such as *COL2A1* and *Aggrecan*, promoting their transcription (Hao et al., 2024). Studies have shown that local injection of *IL-4* in cartilage defect models significantly increases the content of type II collagen and decreases the expression of type I collagen in regenerated cartilage, reducing the formation of fibrocartilage (Hao et al., 2024).
- In immune cells: The *IL-4*/*IL-4R* pathway further strengthens the repair-permissive microenvironment via inhibiting the secretion of *TNF-α* and IL-6 by “inflammation-regulating” macrophages, forming a positive cycle of “immunosuppression-chondrogenic differentiation” (Callejas et al., 2025). Notably, *IL-4* also reduces cartilage matrix degradation via inhibiting the expression of matrix metalloproteinases (MMPs) such as *MMP-3*, *MMP-13*, and *ADAMTS5* (a disintegrin and metalloproteinase with thrombospondin motifs 5), thereby playing a protective role in inflammation-driven cartilage degeneration. This mechanism enables *IL-4* to not only promote cartilage regeneration but also inhibit the progression of cartilage injury, providing a dual-action target for clinical treatment.
- In endothelial cells: Binding of *IL-4* to *IL-4R* inhibits endothelial cell inflammatory responses via activating the JAK1/STAT6 pathway, downregulating the expression of *ICAM-1* and *VCAM-1*, and reducing immune cell adhesion and infiltration. Simultaneously, it promotes endothelial cell secretion of *VEGF*, enhancing angiogenesis ability and maintaining vascular network stability. Studies have found that *IL-4* pretreatment significantly improves the survival efficiency of endothelial cells in the inflammatory microenvironment, with significantly enhanced tube formation ability.

#### 3.2.2 *TGF-β*/Smad pathway: “regulatory hub” of osteogenic/chondrogenic balance, “Stabilizer” of the immune microenvironment, and “promoter” of vascular maturation

Transforming growth factor β (*TGF-β*) is a multifunctional cytokine mainly secreted by “factor-secreting” macrophages, osteoblasts, chondrocytes, and endothelial cells. Its receptors *TGF-βRI/II* are highly expressed on MSCs, immune cells, and endothelial cells. The secretion of *TGF-β* is also regulated by neural signals: Norepinephrine released by sympathetic nerves promotes the expression and secretion of *TGF-β* via activating β2 receptors on macrophages.

- In stem cells: Binding of *TGF-β* to its receptor activates the *Smad2/3* signaling pathway, with its regulatory effects exhibiting significant tissue specificity: In bone regeneration, the *TGF-β*/Smad pathway upregulates the expression of *Runx2*, promoting MSCs differentiation into osteoblasts. In cartilage regeneration, this pathway enhances the matrix synthesis ability of chondrocytes via synergizing with *Sox9* (Thielen et al., 2022).
- In immune cells: *TGF-β* maintains the stability of the regenerative microenvironment via inhibiting the *NF-κB* pathway and reducing the release of inflammatory factors (Jimi et al., 2019). Notably, the concentration of *TGF-β* significantly affects its function: Low concentrations of *TGF-β* mainly promote stem cell proliferation, while high concentrations significantly enhance their differentiation ability (Ling and Robinson, 2002).
- In endothelial cells: *TGF-β* promotes the interaction between endothelial cells and pericytes via the *Smad2/3* pathway, upregulating the expression of vascular smooth muscle cell markers (e.g., α-SMA), and promoting vascular maturation and stability. Simultaneously, *TGF-β* inhibits excessive proliferation and migration of endothelial cells, avoiding abnormal vascular proliferation and maintaining the structural integrity of the vascular network. In bone defect models, *TGF-β* knockout mice exhibit significantly reduced vascular maturation and impaired osteogenic outcomes, while supplementation with exogenous *TGF-β* restores the synergy between vascularization and bone regeneration.

The regulatory role of the *TGF-β*/Smad pathway in bone/cartilage regeneration has been confirmed by numerous basic studies: For example, *TGF-β*1 directly binds to the *Runx2* promoter region via Smad3, enhancing its transcriptional activity. In cartilage development, the *TGF-β*/Smad pathway synergizes with the Hedgehog pathway to regulate chondrocyte proliferation and hypertrophic differentiation. Clinical studies have also found that the level of *TGF-β* in local tissues of patients with bone/cartilage injuries is significantly reduced, and exogenous supplementation of *TGF-β* improves repair outcomes. Additionally, *TGF-β* regulates the function of immune cells, such as promoting Treg cell proliferation and “factor-secreting” macrophage polarization, forming a closed loop of “immune regulation-tissue regeneration-vascular maturation”.

#### 3.2.3 *TNF-α*/*NF-κB* pathway: “initiator” of early inflammation, “regulator” of network balance, and “activator” of early endothelial activation

Tumor necrosis factor α (*TNF-α*) is a core pro-inflammatory factor secreted by “inflammation-regulating” macrophages, with its receptors TNFR1/2 expressed on MSCs, immune cells, and endothelial cells. The expression and function of *TNF-α* are finely regulated by neural signals: In the early stage of injury, neuropeptides such as SP promote the moderate release of *TNF-α*, initiating “beneficial inflammation.” In the later stage, neuropeptides such as CGRP and *NGF* inhibit the excessive expression of *TNF-α*, avoiding prolonged inflammation (Xu et al., 2024).

- In immune cells: In the early stage of injury, binding of *TNF-α* to TNFR promotes the activation of “inflammation-regulating” macrophages via activating the *NF-κB* pathway, releasing more inflammatory factors and accelerating the clearance of necrotic tissue (Xu et al., 2024).
- In stem cells: However, the sustained action of *TNF-α* inhibits stem cell differentiation: Studies confirm that high concentrations of *TNF-α* downregulate the expression of *Runx2* and *Sox9*, while upregulating the expression of *MMP-9*, inhibiting MSCs osteogenic/chondrogenic differentiation and promoting extracellular matrix degradation (Huang et al., 2011). Thus, during regeneration, the expression of *TNF-α* must be strictly regulated by neural signals within the range of “beneficial inflammation,” and its dynamic balance is a key link in the stable function of the neuro-immune regulatory axis (Xu et al., 2024).
- In endothelial cells: *TNF-α* promotes endothelial cells to secrete chemokines such as IL-6 and IL-8 via activating the *NF-κB* pathway in the early stage, upregulating the expression of *ICAM-1* and *VCAM-1*, mediating immune cell adhesion and infiltration, and participating in the construction of the early inflammatory microenvironment. However, high concentrations or sustained presence of *TNF-α* impairs endothelial cell function, leading to increased vascular permeability and vascular network destruction, thus its role is also time-dependent. Additionally, *TNF-α* participates in the temporal regulation of angiogenesis via regulating the secretion of *VEGF* by endothelial cells, promoting vascular sprouting in the early stage and inhibiting vascular maturation in the later stage.

Furthermore, *TNF-α* is involved in bidirectional interactions between chondrocytes and osteoblasts: During endochondral ossification, increased *TNF-α* levels and decreased *Sox9* expression synergistically promote the transdifferentiation of chondrocytes toward osteoblast-like phenotypes, a mechanism particularly important in the cartilaginous callus stage of fracture healing. Meanwhile, the *TNF-α*/*NF-κB* pathway regulates the permeability of vascular endothelial cells, affecting inflammatory cell infiltration and nutrient transport, indirectly participating in the regulation of the regenerative microenvironment. (The local cellular niche for survival and crosstalk during osteochondral injury repair at the cartilaginous callus stage of fracture healing.).

### 3.3 Endothelial cell-mediated core regulatory pathways: “Key link” of osteogenic-immune synergy

Endothelial cells directly regulate the functions of osteoblasts and immune cells via specific signaling pathways, serving as the key link connecting angiogenesis, bone regeneration, and immune regulation. These pathways mainly include the *VEGF*/*VEGF*R, Notch, and *PDGF*/*PDGF*R pathways.

#### 3.3.1 *VEGF*/*VEGF*R pathway: “core pathway” of angiogenic-osteogenic synergy

*VEGF* is a key pro-angiogenic factor secreted by endothelial cells, with its receptors *VEGFR1* and *VEGFR2* widely expressed on endothelial cells, MSCs, and macrophages. The core function of this pathway is to regulate the synergy between angiogenesis and osteogenesis:

- In endothelial cells: Binding of *VEGF* to *VEGFR2* promotes endothelial cell proliferation, migration, and tube formation via activating the *PI3K*/*Akt* and *MAPK* pathways, serving as the core driver of angiogenesis.
- In stem cells: *VEGF* acts on *VEGFR1* on MSCs via paracrine signaling, activating downstream signaling pathways, promoting MSCs differentiation into osteoblasts, and improving MSCs survival efficiency.
- In immune cells: *VEGF* promotes macrophage polarization toward “vascular-associated” subsets, enhancing their synergy with endothelial cells and promoting vascular maturation.

Studies confirm that abnormal activation or inhibition of the *VEGF*/*VEGF*R pathway leads to bone regeneration disorders: Overexpression of *VEGF* results in excessive vascular proliferation and reduced bone mass, while insufficient *VEGF* leads to delayed vascularization and insufficient osteogenesis. Thus, the regulation of this pathway requires maintaining a precise balance.

#### 3.3.2 Notch pathway: “regulatory pathway” of vascular maturation and osteogenic stability

The Notch pathway mainly achieves regulation via direct cell-cell contact: *Jagged1* and Delta-like ligands on endothelial cells bind to the *Notch1* receptor on MSCs and pericytes, activating downstream signaling pathways:

- In the vascular system: The Notch pathway inhibits excessive endothelial cell proliferation, promotes pericyte recruitment and vascular wall maturation, maintaining the stability of the vascular network.
- In stem cells: The Notch signal synergizes with the BMP signal to regulate MSCs differentiation into osteoblasts, while inhibiting their differentiation into adipocytes, maintaining the stability of the osteogenic microenvironment.

#### 3.3.3 *PDGF*/*PDGF*R pathway: “enhancement pathway” of vascular-osteogenic synergy

*PDGF* is mainly secreted by endothelial cells and “vascular-associated” macrophages, with its receptors *PDGFRα* and *PDGFRβ* expressed on pericytes and MSCs:

- In the vascular system: *PDGF*-BB binds to *PDGFRβ* on pericytes, promoting pericyte proliferation and coverage on the endothelial cell surface, enhancing vascular wall stability and integrity.
- In stem cells: *PDGF*-BB promotes MSCs proliferation and migration, while synergizing with *BMP-2* to enhance MSCs osteogenic differentiation potential.

### 3.4 Cross-regulatory pathways: “signal integration center” of the neuro-immune-vascular-stem cell network

The *PI3K*/*Akt* and *MAPK* pathways are core cross-regulatory pathways in the “neuro-immune-vascular-stem cell” network. Both can be activated by neural signals, immune signals, and endothelial signals simultaneously, regulating cell proliferation, differentiation, apoptosis, and functional activation via integrating multi-source signals, serving as the “bridge” connecting the four components.

The activation of the *PI3K*/*Akt* pathway mainly depends on the combined action of neuropeptides (e.g., CGRP), immune factors (e.g., *IL-4*), and endothelial factors (e.g., *VEGF*): CGRP activates *PI3K* via *CRLR*, promoting *Akt* phosphorylation. *IL-4* activates the downstream *PI3K*/*Akt* pathway via *IL-4R*. *VEGF* activates the *PI3K*/*Akt* pathway via *VEGFR2*. The three synergistically upregulate the expression of the anti-apoptotic gene *Bcl-2*, reducing the apoptosis of MSCs and endothelial cells (Liu et al., 2024). Simultaneously, phosphorylated *Akt* enhances MSCs osteogenic/chondrogenic differentiation potential via regulating the activity of *Runx2* and *Sox9* (Klampfleuthner et al., 2022). In bone defect models, inhibition of the *PI3K*/*Akt* pathway leads to a significant decrease in MSCs survival rate, reduced osteogenic differentiation efficiency, insufficient vascular maturation, and weakened mechanical properties of regenerated bone [Tan et al., 2024].

The *MAPK* pathway includes three branches: ERK1/2, JNK, and p38. The ERK1/2 branch mainly mediates the regulatory effects of neural signals and endothelial signals, while the p38 branch mainly responds to immune signals. For example, the *NGF*/*TrkA* pathway activates ERK1/2, promoting MSCs osteogenic differentiation and endothelial cell proliferation. The *VEGF*/*VEGFR2* pathway activates ERK1/2, strengthening endothelial cell tube formation. IL-1β regulates macrophage polarization via activating the p38 pathway [Zhang Z. et al., 2023]. Additionally, the *MAPK* pathway forms cross-links with the *PI3K*/*Akt* pathway, jointly regulating network balance. For example, the activation of ERK1/2 enhances the phosphorylation level of *Akt*, further strengthening its anti-apoptotic and pro-differentiation effects (Carón et al., 2005).

## 4 Application Bre*Akt*hroughs of cutting-edge technologies in network mechanism research (including endothelial cell-related research progress)

The regulation of the “neuro-immune-stem cell” network exhibits high cellular heterogeneity and spatiotemporal specificity. Traditional technologies such as bulk sequencing and immunohistochemistry are difficult to accurately resolve the functional state of individual cells and intercellular interaction patterns. In recent years, the development of cutting-edge technologies such as single-cell omics, organoid models, *in vivo* imaging, new approach methodologies (NAM), microphysiological systems (MPSs), and biosensor-integrated platforms has provided powerful tool support for in-depth exploration of the network’s regulatory mechanisms. Particularly, bre*Akt*hroughs have been made in analyzing the interaction mechanisms between endothelial cells and other cells, as well as the synergy rules between angiogenesis and osteogenesis, promoting research from the “population level” to the “single-cell level” and “dynamic level.”

### 4.1 Single-cell omics: “Golden tool” for resolving endothelial cell heterogeneity and multi-cell interactions

Single-cell RNA sequencing (scRNA-seq) technology can resolve gene expression profiles at the single-cell level, clarify the functional states and differentiation trajectories of different cell subsets, and provide direct evidence for breaking the traditional M1/M2 classification framework and revealing the cellular basis of the neuro-immune-vascular regulatory axis.

In terms of resolving immune cell heterogeneity, scRNA-seq studies have found that macrophages in the bone defect microenvironment are not simply divided into M1 and M2 types but exist as multiple functionally distinct subsets, such as “inflammation-regulating,” “factor-secreting,” “phagocytic,” and “vascular-associated” (Li J. et al., 2025). Among these, “factor-secreting” macrophages highly express repair factors such as *IL-4* and *TGF-β*, with their proportion positively correlated with stem cell osteogenic differentiation efficiency. “Inflammation-regulating” macrophages highly express CGRP receptor (*CRLR*) and adrenergic receptor (ADRB2), directly responding to neural signal regulation and maintaining the dynamic balance of the inflammatory microenvironment via rapid functional switching (Wang et al., 2024). These findings break the traditional macrophage classification model, reveal the cell specificity of the neuro-immune regulatory axis, and provide new targets for precise targeted regulation of macrophage subsets. Additionally, scRNA-seq has found that neutrophils and T cell subsets also exhibit functional heterogeneity, with some subsets participating in neuro-immune regulation via expressing neuropeptide receptors, further enriching the cellular composition of the neuro-immune regulatory axis.

In terms of resolving endothelial cell heterogeneity, scRNA-seq studies have revealed three functionally distinct subsets of endothelial cells in the bone regeneration microenvironment: “proliferative endothelial cells” (highly expressing Ki67, *VEGFR2*), “mature endothelial cells” (highly expressing PECAM-1, *eNOS*), and “inflammation-regulating endothelial cells” (highly expressing *ICAM-1*, IL-6). Among these, “proliferative endothelial cells” mainly exist in the inflammatory and proliferative phases, responsible for angiogenesis. “Mature endothelial cells” exist in the remodeling phase, maintaining vascular stability. “Inflammation-regulating endothelial cells” regulate immune cell infiltration via secreting IL-6 and *CXCL8*, serving as the key subset connecting vascular and immune regulation. Additionally, scRNA-seq has found that the gene expression profiles of “vascular-associated” macrophages and “mature endothelial cells” are highly synergistic, with both highly expressing the same cell adhesion molecules (e.g., *VCAM-1*) and growth factors (e.g., *PDGF*-BB), providing molecular evidence for their direct interaction.

In terms of resolving stem cell differentiation trajectories, single-cell pseudotime analysis has clarified the differentiation path of MSCs during bone/cartilage regeneration: MSCs first differentiate into “pre-osteoblasts/pre-chondrocytes,” which highly express surface markers such as CD105 and CD73, while expressing neurotransmitter receptors and immune factor receptors, serving as direct “responders” to neuro-immune signals. Subsequently, “pre-osteoblasts/pre-chondrocytes” differentiate into mature osteoblasts or chondrocytes under the regulation of different neuro-immune signal combinations (Yu et al., 2024). Additionally, single-cell pseudotime analysis has revealed that the differentiation timing of “pre-osteoblasts” and “mature endothelial cells” is highly synchronized, with their gene expression profiles showing significant correlation during the remodeling phase, such as simultaneous high expression of *Runx2* and *VEGFR2*, confirming the synergy between osteogenesis and vascular maturation. Furthermore, scRNA-seq has found that the differentiation direction of MSCs is closely related to the combination mode of neuro-immune-vascular signals—the CGRP + *IL-4*+*VEGF* signal combination induces their differentiation into chondrocytes, while the *NGF* + low-concentration *TNF-α*+*PDGF* signal combination promotes their differentiation into osteoblasts (Huang et al., 2011).

Single-cell ATAC-seq (scATAC-seq) technology can reveal the regulatory effects of neuro-immune-vascular signals on the epigenetic state of stem cells via resolving chromatin accessibility. Studies have found that CGRP enhances the transcriptional activity of *Runx2* via regulating the chromatin accessibility of the *Runx2* gene promoter region, thereby promoting MSCs osteogenic differentiation (Hojo et al., 2022). Additionally, *VEGF* enhances the expression of *BMP-2* via regulating the chromatin accessibility of the *BMP-2* gene promoter region in MSCs, synergistically promoting osteogenic differentiation, revealing the mechanism of endothelial signal regulation of stem cell differentiation from the epigenetic level.

### 4.2 Organoid models: constructing a “3D microenvironment” for neuro-immune-vascular-stem cell interactions

Organoids are 3D cellular structures self-assembled by stem cells *in vitro*, with cellular composition and structural characteristics similar to *in vivo* tissues. They can simulate the dynamic interaction process of the *in vivo* neuro-immune-vascular regulatory axis, serving as an ideal model for studying network regulatory mechanisms and drug screening.

Currently, the construction of bone/cartilage organoid models has achieved “multi-cell co-culture,” i.e., co-seeding MSCs, macrophages, nerve cells (or neurospheres), and endothelial cells in biomimetic scaffold materials, simulating the *in vivo* regeneration process via regulating culture conditions (Li et al., 2019). The advantages of such models include: ① Precise control of the type and concentration of neuro-immune-vascular signals, clarifying the effects of single signals (e.g., CGRP, *VEGF*) on immune cell subset polarization, endothelial cell tube formation, and stem cell differentiation. ② Dynamic observation of the interaction process among the four components, such as the contact between nerve terminals and “inflammation-regulating” macrophages, the tube formation between “vascular-associated” macrophages and endothelial cells, and the paracrine signal transmission between “factor-secreting” macrophages and stem cells (Zhao et al., 2025). However, traditional organoid models have significant limitations—lack of mechanical stimulation required for bone/cartilage tissues (e.g., synovial fluid pressure, bone tissue stress), while mechanical force is a key factor regulating the phenotypes of chondrocytes, osteocytes, immune/nerve cells, and endothelial cells. To address this deficiency, researchers have begun combining organoids with microfluidic technology, simulating the *in vivo* mechanical environment via fluid shear stress, significantly improving the functional maturity of organoids.

For example, researchers using a bone organoid model (containing MSCs, macrophages, neurospheres, and endothelial cells) found that simultaneous addition of CGRP, *IL-4*, and *VEGF* significantly increases the proportion of “factor-secreting” macrophages and “mature endothelial cells” in organoids, with vascular density and mineralization degree significantly higher than those in groups treated with any single factor alone. The mechanical properties of organoids are more similar to *in vivo* bone tissue. This result confirms the mechanism of neuro-immune-vascular signal synergistic regulation of osteogenesis, providing a basis for the development of multi-factor synergistic biomaterials. In cartilage organoid models, the addition of *NGF* and *VEGF* significantly inhibits the expression of type I collagen and promotes the secretion of type II collagen in organoids, while upregulating the proportion of “factor-secreting” macrophages and “mature endothelial cells,” indicating that *NGF* and *VEGF* can indirectly maintain the chondrocyte phenotype via regulating the immune microenvironment and vascular network (Wang M. et al., 2025). Additionally, organoid models can be used for drug screening—for example, via screening small-molecule compounds that promote “factor-secreting” macrophage polarization and endothelial cell tube formation, curcumin was found to enhance the regenerative capacity of cartilage organoids via activating the CGRP/*CRLR* pathway and *VEGF*/*VEGFR2* pathway (Zhu et al., 2020).

**FIGURE 2:**
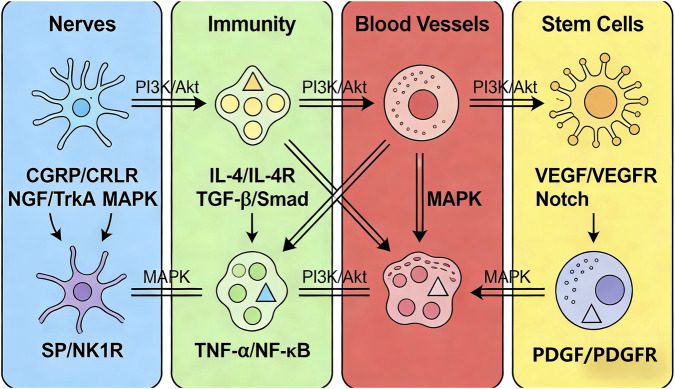
Immunofluorescence staining of frozen sections of bone/cartilage organoids co-cultured with MSCs, macrophages, nerve terminals and endothelial cells. CD31 (green) labels endothelial cells forming vascular-like structures, CGRP (red) labels nerve terminals, CD206 (cyan) labels “factor-secreting” macrophages, and COL2A1/Runx2 (magenta) labels chondrocytes/osteoblasts. DAPI (blue) stains cell nuclei. The results show the spatial distribution and close contact of nerve, immune, vascular and stem cells in the 3D organoid microenvironment, reflecting the in vitro simulation of the neuro-immune-vascular-stem cell crosstalk by bone/cartilage organoids.

The recently developed “vascularized bone organoid” model further simulates the synergy between angiogenesis and osteogenesis *in vivo*: In this model, endothelial cells can spontaneously form functional vascular networks, providing nutrient supply to the interior of organoids, significantly improving the survival time and mineralization degree of organoids. When the *VEGF* gene in endothelial cells is knocked out, the vascular density and osteogenic differentiation efficiency of organoids are significantly reduced, confirming the core role of endothelial cells in organoid maturation (Figure 2).

### 4.3 *In vivo* imaging technology: dynamic tracking of the “spatiotemporal rules” of neuro-immune-vascular regulation

*In vivo* imaging technologies (e.g., two-photon microscopy) can dynamically track the interaction process of the “neuro-immune-vascular-stem cell” network in live animal models, clarifying the spatiotemporal specificity of the neuro-immune-vascular regulatory axis, and making up for the deficiencies of *in vitro* experiments and static section analysis.

Two-photon microscopy, with its deep tissue imaging capability, can real-time observe the extension of nerve terminals, recruitment of immune cells, formation of endothelial cell blood vessels, and differentiation of stem cells in bone defect models. Studies using sensory nerve mice expressing GFP, macrophages expressing RFP, endothelial cells expressing CFP, and MSCs expressing YFP found via two-photon imaging: 3 days after injury, sensory nerve terminals begin to extend toward the injury site, endothelial cells initiate vascular sprouting, and their extension direction is highly consistent with the recruitment direction of “inflammation-regulating” macrophages. 7 days after injury, nerve terminals form close contact with “factor-secreting” macrophages, endothelial cells form initial vascular plexuses, and “vascular-associated” macrophages distribute around blood vessels. 14 days after injury, mature vascular networks are formed, and MSCs differentiate into osteoblasts around blood vessels, confirming the temporal synergy rule of “neuro-immune-vascular-stem cell”. This finding confirms the dual mode of “contact-dependent regulation” and “paracrine regulation” among the four components, supplementing the traditional single regulatory theory and providing a new perspective for the mechanism research of the neuro-immune-vascular regulatory axis.

### 4.4 Application of new approach methodologies (NAM), microphysiological systems (MPSs) and biosensor integrated platforms

Under the framework of New Approach Methodologies (NAM), pharmacological and genetic tools targeting specific kinases provide powerful means for the precise dissection of key nodal functions within the “neuro-immune-vascular-stem cell” network. For instance, to elucidate the specific role of *PI3K*/*Akt* signaling in cell fate determination, researchers frequently combine small-molecule kinase inhibitors with RNA interference (RNAi) technology. In relevant studies, applying *PI3K* inhibitors such as LY294002 or small interfering RNA (siRNA) targeting the *PI3K* p85 subunit can effectively block pathway activity, thereby establishing its necessity in regulating cell survival. Similarly, the use of PDK-1 inhibitors like OSU-03012 or corresponding siRNA enables specific perturbation of PDK-1 function, revealing its distinct impact on downstream *Akt* activation and cellular radiosensitivity. The application of these NAMs makes it possible to achieve spatiotemporally specific manipulation and functional validation of individual targets within a complex network, laying the groundwork for the subsequent development of precise targeted regulatory strategies.

Microphysiological systems (MPSs), also known as “organ-on-a-chip,” can precisely simulate the physiological microenvironment of bone/cartilage *in vitro* via integrating microfluidics, biomaterials, and cell co-culture technology, including mechanical stimulation, vascular perfusion, and neuro-immune-vascular-stem cell interactions. Compared with traditional organoids, MPSs have the following advantages: ① Simulation of multi-tissue synergy (e.g., combined models of bone-cartilage-synovium-vascular). ② Precise regulation of physical signals such as mechanical force and fluid shear stress. ③ Realization of long-term dynamic culture and real-time monitoring. For example, bone-cartilage MPSs can simulate the pressure environment and nutrient exchange process of the joint cavity. In this system, CGRP released by nerve terminals can be directionally transported to the injury site via blood flow, regulating macrophage polarization, endothelial cell tube formation, and MSCs differentiation, with a regulatory mode more similar to *in vivo*. Additionally, the “vascularized bone-on-a-chip” model can simulate the hemodynamic environment of bone marrow microvessels via microfluidic technology. Endothelial cells form functional vascular networks in vascular channels, synergizing with surrounding MSCs and macrophages, with osteogenic efficiency and vascular maturity significantly higher than traditional static culture models.

As a vital complement to NAM and microphysiological systems (MPSs), integrated biosensor platforms further enhance the precision and real-time performance of simulating complex physiological microenvironments, deciphering target functions, and evaluating regulatory effects. By the seamless integration of high-sensitivity biosensors (e.g., electrochemical, optical, and piezoelectric biosensors) with MPSs systems, this platform enables real-time, dynamic, and high-throughput monitoring of key indicators within the neuro-immune-vascular-stem cell network, including the concentrations of bioactive molecules (e.g., cytokines, growth factors, and neurotransmitters), cellular electrophysiological activities, and extracellular matrix remodeling. For instance, the integration of electrochemical biosensors into osteochondral MPSs allows for real-time detection of the dynamic secretory profiles of molecules such as calcitonin gene-related peptide (CGRP) and tumor necrosis factor-α (*TNF-α*), thus realizing precise quantification of the effects of NAM-mediated regulation (e.g., *PI3K*/*Akt* signaling pathway inhibition) on neuro-immune crosstalk. In addition, the coupling of optical biosensors with vascularized bone chips facilitates real-time tracking of vascular endothelial growth factor (*VEGF*) concentration gradient changes during endothelial cell angiogenesis, which provides direct experimental evidence for assessing the vascular regulatory efficacy of NAM-targeted strategies.

The synergistic application of NAM, MPSs, and integrated biosensor platforms constructs a complete research framework characterized by *precision modulation - in vitro simulation - real-time monitoring*. Specifically, NAM provides a precise regulatory approach for targeting specific functional nodes, MPSs recapitulate in vivo-like complex physiological microenvironments, and integrated biosensor platforms achieve real-time quantitative monitoring of regulatory outcomes. These three components mutually support and complement each other’s inherent advantages: they not only overcome the limitations of traditional research methods that fail to mimic complex *in vivo* interplays and capture dynamic regulatory processes in real time, but also offer a novel technical avenue for mechanistic investigations into the neuro-immune-vascular-stem cell network and the development of precision targeted regulatory strategies. Collectively, this integrated technological system markedly elevates the scientific rigor and translational value of relevant research.

### 4.5 Summary of advantages, disadvantages, and challenges of cutting-edge technologies

Table 1 shows a summary of the advantages, disadvantages, and challenges of cutting-edge technologies.

**Table 1:** Summary of advantages, disadvantages, and challenges of cutting-edge technologies.

Technology method	Core advantages	Limitations	Current challenges
Single-cell omics (scRNA-seq/scATAC-seq)	Resolves immune cell and endothelial cell heterogeneity; identifies cellular targets of neuro-immune-vascular regulation; reveals epigenetic regulation	High cost; complex data analysis; inability to track dynamically	Integrate multi-omics data (transcriptome + epigenome + proteome + spatial omics); realize dynamic monitoring of neuro-immune-vascular signals at the single-cell level
Organoid models	Simulates neuro-immune-vascular-stem cell interactions in 3D structure; applicable for drug screening	Lack of mechanical stimulation; difficulty in simulating multi-tissue synergy; limited functional maturity	Combine mechanical loading and microfluidic technology; construct “patient-specific” organoids with functional vascular networks; precisely simulate the neuro-immune-vascular regulatory axis
In vivo imaging technology	Dynamically tracks spatiotemporal rules of neuro-immune-vascular-stem cell interactions; non-invasive monitoring	Limited imaging depth; resolution affected by tissue penetration	Develop high-specificity fluorescent probes for neuropeptides/immune factors/VEGF; improve deep tissue imaging resolution; realize real-time dynamic tracking of interactions among the four components
New approach methodologies (NAM)	High-throughput screening of neuro-immune-vascular regulatory targets; artificial intelligence prediction of interaction relationships	Dependent on large amounts of data support; prediction results require experimental verification	Establish standardized data processing procedures; improve the accuracy and reproducibility of neuro-immune-vascular signal interaction prediction
Microphysiological systems (MPSs)	Simulates physiological microenvironment (mechanical + nutritional + multi-tissue); real-time monitors neuro-immune-vascular signals	Complex manufacturing process; difficulty in large-scale application	Simplify preparation procedures; realize multi-organ chip combination; simulate the synergistic regulation of neuro-immune-vascular-stem cell; construct “vascularized” organ chips

## 5 Discussion

### 5.1 Core insights and research value of network regulatory mechanisms: the central role of the neuro-immune-vascular regulatory axis

The proposal of the “neuro-immune-stem cell” cross-system regulatory network breaks the traditional thinking of “isolated viewing of cell functions” in bone/cartilage regeneration research. The addition of endothelial cells further improves this network, revealing that regeneration is a complex biological event involving the synergy of four systems: nerves, immunity, blood vessels, and stem cells. Its core insight is: Neural signals are the “initiating switch” of regeneration and the “shaper” of the immune microenvironment. Immune cells are the “responders” to neural signals and the “regulators” of stem cell functions. Stem cells are the “final executors” of tissue repair. The vascular system (centered on endothelial cells) is the “key carrier” of nutrient supply and signal transmission. The four components jointly determine the efficiency and quality of regeneration via precise temporal interactions and signal cross-talk. Among these, the neuro-immune-vascular regulatory axis is the core hub of the network. Neural signals indirectly regulate stem cell functions via regulating the recruitment and polarization of immune cells, as well as the activation and angiogenesis of endothelial cells. This regulatory mode is more common and critical than direct regulation of stem cells by single signals (Dalle Carbonare et al., 2025; Park et al., 2024).

The clarification of this mechanism provides a new theoretical framework for bone/cartilage regeneration research—future intervention strategies need to shift from “targeting single targets” to “regulating network balance,” with particular focus on optimizing the neuro-immune-vascular regulatory axis. For example, via synergistically regulating neuropeptide secretion, macrophage subset polarization, endothelial cell angiogenesis, and stem cell differentiation, a suitable microenvironment for regeneration is created, rather than simply pursuing high stem cell differentiation efficiency.

### 5.2 Current bottlenecks and controversies in research

Despite significant progress in the research of the “neuro-immune-vascular-stem cell” network, numerous bottlenecks and controversies remain, restricting the process of translating basic research into clinical applications.

First, the limitations of the traditional M1/M2 macrophage classification framework have become an important obstacle to research precision. Although this framework is still widely used in biomaterials research, single-cell omics studies have confirmed that it cannot cover the heterogeneous characteristics of immune cells, ignoring the existence of intermediate phenotypes and functional subsets. Meanwhile, research on endothelial cell heterogeneity is still in its infancy, with the markers and regulatory mechanisms of different functional subsets (e.g., “proliferative,” “mature,” “inflammation-regulating”) not yet fully clarified, lacking unified classification standards, which affects the precision of endothelial cell-targeted strategies. Future research needs to refer to single-cell atlas analysis results, adopt more precise classification schemes for immune cells and endothelial cells, clarify the role of each functional subset in the neuro-immune-vascular regulatory axis, and avoid mechanism analysis errors caused by classification biases.

Second, the problem of spatiotemporal specificity of regulatory targets has not been solved. The roles of neural signals, immune factors, and vascular factors are strictly concentration-dependent and time-window-dependent. For example, low concentrations of *TNF-α* can initiate beneficial inflammatory responses, while high concentrations inhibit stem cell differentiation. CGRP can promote immune cell recruitment and endothelial cell activation in the early stage of injury, but may lead to excessive neural ingrowth in the later stage, affecting cartilage function (). *VEGF* can promote vascular sprouting in the early stage, while overexpression leads to abnormal vascular proliferation and reduced bone mass. Currently, there is a lack of technical means to precisely regulate the spatiotemporal characteristics of target expression. How to achieve “releasing the correct concentration of signaling molecules at the right time and right location” is the core obstacle to clinical translation.

Third, the *in vitro* simulation of complex microenvironments is insufficient. Existing organoid models and MPSs can partially simulate the *in vivo* microenvironment, but it is difficult to fully reproduce the dynamic changes of mechanical stimulation, 3D network of innervation, dynamic infiltration of immune cells, and maturation process of endothelial cell vascular networks. For example, *in vitro* cultured bone organoids cannot simulate the synergistic regulatory effect of sympathetic and sensory nerves *in vivo*, and the vascular networks formed by endothelial cells lack pericyte coverage, with insufficient stability, leading to differences in regeneration outcomes from *in vivo* (Hong et al., 2025). Additionally, there are individual differences in the neuro-immune-vascular status of different patients. How to construct “patient-specific” network models is also a problem that needs to be solved for personalized treatment.

Finally, some core mechanisms are controversial. For example, regarding the role of nerve growth factor (*NGF*) in cartilage regeneration, some studies believe that *NGF* can promote CPC differentiation, while others find that *NGF* induces neural fiber invasion into cartilage tissue, accelerating the progression of osteoarthritis (Wang M. et al., 2025). Controversies also exist regarding the application dosage and timing of *VEGF*: Some studies believe that early high-dose *VEGF* can rapidly initiate angiogenesis and promote osteogenesis, while others find that high-dose *VEGF* leads to excessive vascular proliferation and inhibits osteoblast function. Such controversies may stem from differences in animal models (young vs. elderly), injury types (acute injury vs. chronic degeneration), intervention concentrations, and timing used in studies, which need to be further verified via standardized experimental designs.

### 5.3 Key challenges in clinical translation

In the process of translating basic research results into clinical applications, there is a practical challenge of “disconnection between mechanism research and clinical needs.” On one hand, most studies are based on young and healthy animal models, while clinical patients are mostly elderly, often accompanied by underlying diseases such as osteoporosis and diabetes, with abnormal neuro-immune-vascular functions, leading to difficulties in reproducing positive results from animal experiments in clinical settings. For example, sympathetic nerve dysfunction in diabetic patients leads to reduced CGRP secretion, impaired macrophage polarization, impaired endothelial cell function, insufficient *VEGF* secretion, and decreased angiogenesis ability. At this time, the therapeutic effect of simple MSCs transplantation is significantly reduced (Li X. et al., 2025). On the other hand, existing intervention methods (e.g., signal factor sustained-release scaffolds) have potential biosafety risks. For example, long-term release of exogenous neuropeptides may cause local pain or inflammatory responses, immunomodulatory drugs may induce systemic immunosuppression, and excessive *VEGF* release may lead to abnormal vascular proliferation and even tumor risks (Zhang et al., 2007).

## 6 Future outlook

Future research on the “neuro-immune-vascular-stem cell” network should focus on the core chain of “mechanism deepening - technological innovation - clinical translation”. Centered on the neuro-immune-vascular regulatory axis and taking the heterogeneity of immune cells and endothelial cells as a bre*Akt*hrough point, bre*Akt*hroughs should be achieved in the following four directions:

First, utilize multi-omics technologies to decipher the precise mechanisms of the neuro-immune-vascular regulatory axis. Combine single-cell omics, spatial transcriptomics, proteomics, and epigenomics technologies to map a three-dimensional “cell-signal-space” regulatory atlas, and clarify the dynamic changes of immune cell subsets, endothelial cell subsets, and the regulatory targets of neural signals under different injury microenvironments. For example, through spatial transcriptomics technology, locate the precise spatial distribution of nerve terminals, “inflammation-regulating” and “factor-secreting” macrophages, different subtypes of endothelial cells, and stem cells at the injury site, and reveal the molecular basis of “contact-dependent regulation” among the four. Meanwhile, use epigenetics technologies (such as ATAC-seq, ChIP-seq) to analyze the regulatory effects of neuro-immune-vascular signals on the epigenetic states of immune cells, endothelial cells, and stem cells, providing targets for the development of epigenetic intervention drugs.

Second, develop smart responsive biomaterials to achieve precise regulation of the neuro-immune-vascular axis. Based on the temporal regulatory rules of the network, design “spatiotemporally responsive” bionic scaffolds. For instance, release calcitonin gene-related peptide (CGRP) and low-dose vascular endothelial growth factor (*VEGF*) in the early stage of injury (inflammatory phase) to recruit “inflammation-regulating” macrophages and initiate vascular sprouting; release interleukin-4 (*IL-4*) and moderate-dose *VEGF* in the middle stage (proliferative phase) to induce the polarization of “factor-secreting” macrophages and vascular maturation; stop factor release in the late stage (remodeling phase) to provide mechanical support and inhibit excessive ingrowth of nerve fibers (Lu et al., 2024). Additionally, combine 3D bioprinting technology to construct personalized scaffolds that simulate the anatomical structure, neural innervation pattern, and vascular network of bone/cartilage, improving the structural and functional matching of regenerated tissues. Furthermore, develop smart materials with mechanical sensing functions to real-time monitor the mechanical properties and vascularization degree of regenerated tissues, and dynamically adjust the release of neuropeptides/immune factors/*VEGF* according to the monitoring results to achieve “on-demand therapy” (Lu et al., 2025).

Third, establish “patient-specific” network regulatory models to promote clinical translation. Based on patients' age, gender, underlying diseases (such as diabetes, osteoporosis), injury types, and neuro-immune-vascular function status (detected by imaging and blood biomarkers), construct personalized “neuro-immune-vascular-stem cell” network models, and screen the optimal intervention plan through organoid technology. For example, for elderly patients with bone defects, whose nerve terminal secretion function and endothelial cell angiogenesis ability are impaired, a combined treatment strategy of “neuropeptide supplementation + *VEGF* sustained release + immune regulation” can be designed; for diabetic patients, it is necessary to first improve their abnormal immune cell polarization and endothelial cell dysfunction before stem cell transplantation (Ma et al., 2025). At the same time, carry out multi-center, small-sample clinical pilot studies to verify the safety and effectiveness of intervention strategies based on the neuro-immune-vascular regulatory axis, laying a foundation for large-scale clinical application.

Fourth, integrate interdisciplinary approaches to solve complex scientific problems. Strengthen the cross-cooperation among neurobiology, immunology, vascular biology, materials science, mechanics, and clinical medicine. For example, use artificial intelligence technology to analyze multi-omics data and predict the interaction relationships between neuro-immune-vascular signals and immune cell subsets, endothelial cell subsets, and stem cells; combine *in vivo* imaging technology and big data analysis to dynamically evaluate the neuro-immune-vascular regulatory status of clinical patients, realizing the dynamic adjustment of treatment plans (Zhang Q. et al., 2023). In addition, establish a standardized research system for the “neuro-immune-vascular-stem cell” network, including unified cell culture standards, animal model evaluation indicators (including vascularization degree assessment), and clinical efficacy evaluation systems, to solve the comparability problem of different research results.

## 7 Conclusion

The cross-boundary regulatory network of “neuro-immune-vascular-stem cell” is the core regulatory mechanism for bone/cartilage regeneration, among which the neuro-immune-vascular regulatory axis plays a leading role: neural signals regulate the recruitment and polarization of immune cell subsets, as well as the activation and angiogenesis of endothelial cells through core pathways such as CGRP/*CRLR*, nerve growth factor (*NGF*)/tropomyosin receptor kinase A (*TrkA*), and substance P (SP)/neurokinin one receptor (*NK1R*); immune cells regulate the proliferation and differentiation of stem cells by secreting cytokines such as *IL-4*, transforming growth factor-β (*TGF-β*), and tumor necrosis factor-α (*TNF-α*); endothelial cells provide nutritional support by constructing functional vascular networks and secrete factors such as bone morphogenetic protein-2 (*BMP-2*) and *VEGF* to synergistically regulate osteogenesis. The four achieve precise temporal interaction and functional synergy through cross-signaling pathways such as phosphatidylinositol 3-kinase (*PI3K*)/protein kinase B (*Akt*) and mitogen-activated protein kinase (*MAPK*).

The application of single-cell omics technology has revealed the high heterogeneity of immune cells and endothelial cells, breaking the traditional cognition of M1/M2 dichotomy and endothelial cell homogeneity, and providing a new perspective for the precise analysis of the neuro-immune-vascular regulatory axis; the development of cutting-edge technologies such as organoid models, *in vivo* imaging, new analytical methods (NAM), and microphysiological systems (MPSs) has provided strong support for in-depth understanding of the spatiotemporal regulatory rules of the network. Although current research faces bottlenecks such as poor spatiotemporal specificity of regulatory targets, insufficient microenvironment simulation, and difficulty in clinical translation, smart biomaterials and personalized intervention strategies developed based on the neuro-immune-vascular regulatory axis have shown broad application prospects.

In the future, through interdisciplinary integration to deepen mechanism research and optimize regulatory strategies, it is expected to realize the leap of bone/cartilage regeneration from “basic experiment” to “clinical cure”, providing more effective treatment plans for patients with bone/cartilage injuries.,
